# Movi 2: fast and space-efficient queries on pangenomes

**DOI:** 10.1093/bioinformatics/btag362

**Published:** 2026-06-17

**Authors:** Mohsen Zakeri, Nathaniel K Brown, Travis Gagie, Ben Langmead

**Affiliations:** Department of Computer Science, Johns Hopkins University, Baltimore, Maryland 21218, United States; Department of Computer Science, Johns Hopkins University, Baltimore, Maryland 21218, United States; Faculty of Computer Science, Dalhousie University, Halifax, Nova Scotia B3H 4R2, Canada; Department of Computer Science, Johns Hopkins University, Baltimore, Maryland 21218, United States

## Abstract

**Summary:**

Space-efficient compressed indexing methods are critical for pangenomics and for avoiding reference bias. In the Movi study, we implemented the move-structure index, highlighting its locality-of-reference and speed. However, Movi had a high memory footprint compared to other compressed indexes. Here, we introduce Movi 2 and describe new methods that greatly reduce size and memory footprint of move structure-based indexes. The most compressed version of Movi 2 reduces the Movi index’s space footprint more than five-fold. We also introduce sampling approaches that enable trade-offs between query and space efficiency. To demonstrate, we show that Movi 2 achieves advantageous time and space tradeoffs when applied to large pangenome collections, including both the first and second releases of the Human Pangenome Reference Consortium (HPRC) collection, the latter of which spans over 460 human haplotypes. We show that Movi 2 dominates prior methods on both speed and memory footprint, including both r-index-based and our previous move-structure-based method.

**Availability and implementation:**

The methods we developed for Movi 2 are publicly available at https://github.com/mohsenzakeri/Movi.

## 1 Introduction

Long-read sequencing has enabled the building of large pangenome collections, such as the Human Pangenome Reference Consortium (HPRC) (Liao *et al.* 2023) which includes 466 human haplotypes in its recent second release. By using pangenome indexes rather than linear references, we can analyze sequencing reads while reducing the reference bias (Pritt *et al.* 2018, Chen *et al.* 2021, Lin *et al.* 2024). Indexes designed for individual reference genomes are not practical for pangenomes since they scale linearly, i.e. like Θ(n) where *n* is the total length of the pangenome collection. Compressed indexes, on the other hand, scale with measures of the non-redundant content in the collection; e.g. the *r*-index (Gagie *et al.* 2018, 2020) scales like Θ(r), where *r* is the number of runs in the Burrows-Wheeler Transform (BWT) (Burrows and Wheeler 1994) of the reference collection. Two compressed index types have proven quite practical: BWT-based full-text compressed indexes, (Kuhnle *et al.* 2020, Nishimoto and Tabei 2021) and *k*-mer indexes which consist of all substrings of length *k* in the reference (*k*-mers). The size of a *k*-mer index grows with the number of distinct *k*-mers in the input, which is related to subword complexity (Raskhodnikova *et al.* 2013, Kociumaka *et al.* 2023). Similar to *r*, it reflects the amount of non-redundant sequence in the reference.

The BWT reversibly permutes the letters of the input according to the alphabetical order of the letters’ right contexts, i.e. the sequence that comes to each letter’s right. This permutation tends to bring equal characters together into long stretches, where a maximal equal-letter substring of the BWT is called a “BWT run,” and the total number of runs in the BWT is called *r*. When the text becomes more repetitive, the runs become longer and fewer in number. As we add more closely related genomes to a pangenome, *n* grows linearly while *r* grows more slowly. The ratio n/r can be considered the compression ratio achieved.

BWT-based indexes are full-text indexes; they allow us to search in the reference for query strings of any length. *k*-mer-based approaches are less flexible and are limited to queries of a fixed length *k*. These approaches are also generally lossy, in that they lose information about continuous sequences longer than *k*. On the other hand, the *k*-mer-based indexes are computationally efficient, making them a practical and widely used method for indexing.

The data structures behind full-text indexes have generally had poor locality-of-reference. That is, when the index is being queried, the steps of the query process require access to multiple components of the data structure residing in different parts of memory, causing delays while the needed memory is fetched (i.e. it incurs “cache misses”). Locality-of-reference is the tendency of an algorithm to access memory locations that are close to one another or that have been accessed recently. Modern hardware is optimized for algorithms with better locality-of-reference, since they keep recently used chunks of data in fast cache memory that resides close to the processor. When accesses are scattered across many distant locations and no one location is accessed frequently (poor locality-of-reference), performance suffers because of the need to repeatedly fetch data from slower main memory. Even algorithms with optimal theoretical time complexity can run slowly in practice if they exhibit poor locality-of-reference. For example, scanning sequentially through a contiguous array is typically much faster than repeatedly jumping between unrelated memory addresses, even if both perform the same number of logical operations.

In the case of algorithms based on the *r*-index, poor locality-of-reference results from the fact that a single step of the query algorithm must access a variety of bitvectors and arrays, as well as the index data structures that accompany them.

Recently Movi (Zakeri *et al.* 2024) was introduced for indexing pangenomes. It is designed based on the move structure (Nishimoto and Tabei 2021). The move structure is also a BWT-based compressed full-text index, but it has high locality-of-reference. This means that its memory accesses tend to be predictable and nearby; as a result, it rarely needs to pause for cache misses. Movi is significantly faster to query compared to other full-text indexes and its query speed is comparable to *k*-mer-based methods.

Similarly to the *r*-index, the move structure grows with the number of runs in the BWT, i.e. like Θ(r). But the number of bytes required per run (the constant factor) is large compared to other full-text approaches. In this work, we present new ideas to compress the Movi index. The main component of the index is a table with height that grows by O(r) and width which is a large constant factor. Each row corresponds to a BWT run and consists of columns that correspond to the run-length representation, the LF-mapping of the run head, and the thresholds information (Bannai *et al.* 2020) which are offsets in the run. We first introduce a splitting approach that minimizes the space required for the storage of thresholds. We further reduce the size of rows with a scheme that splits rows in order to cap their length. Finally, we present methods for reducing the space required to store the ξ (also called *id*) component of the structure, which holds the indexes of the “destination” Movi row containing the LF-mapping of the current run’s head. This value needs ${\;\text{log}\;}_{2}(r)$ bits by default, however, we present two approaches for its compression. These ideas exploit the monotonic growth of the values per character, and the relationship between the rows to either sample these values, or use a blocking scheme to encode differences relative to larger components.

Movi 2 is capable of building pangenome indexes that are significantly smaller than Movi’s, while also supporting faster queries. As a demonstration, we measure Movi 2’s index size and speed for three datasets: a set of 94 assembled haplotypes from the HPRCv1 (Liao *et al.* 2023) release, a larger set of 466 haplotypes from the HPRCv2 release (Liao *et al.* 2023), and a set of 7692 complete genomes from 7 bacterial species. We show that the trade-off between index size and query speed is superior using these new approaches compared to either SPUMONI (based on the *r*-index), or to the prior version of Movi. Finally, we explore how Movi 2’s prefetching combines with thread parallelization to achieve further speed gains.

## 2 Materials and methods

### 2.1 Preliminaries

#### 2.1.1 The Burrows Wheeler transform

The Burrows Wheeler transform (BWT) of *T* is a reversible transformation that permutes *T*’s characters according to the lexicographical order of their right contexts (Burrows and Wheeler 1994). Let *T* be a string of length *n*, |T|=n, over the alphabet Σ and a special terminal character $∉Σ such that ∀c∈Σ:$<c. *T[i]* denotes the character at 1-based-offset *i* in T and T[i  ..  n] denotes a suffix of T starting at position *i*. L=BWT(T) is a permutation of all the characters in *T* such that for all *i* and *j* such that 1≤i,j≤n, we have L[j]=T[i] if and only if the suffix T[i+1  ..  n] is the *j*th smallest suffix of *T* in lexicographic order.

When there are repetitions in *T*, right contexts also repeat, leading to contiguous segments (or “runs”) of identical characters in the BWT. The run-length encoded BWT (RLBWT) compresses a run to a pair consisting of the character and the run length. RLBWTis used to build effective compressed indexes for pangenomes (MäKinen and Navarro 2005, Mun *et al.* 2020).

**Figure 1:**
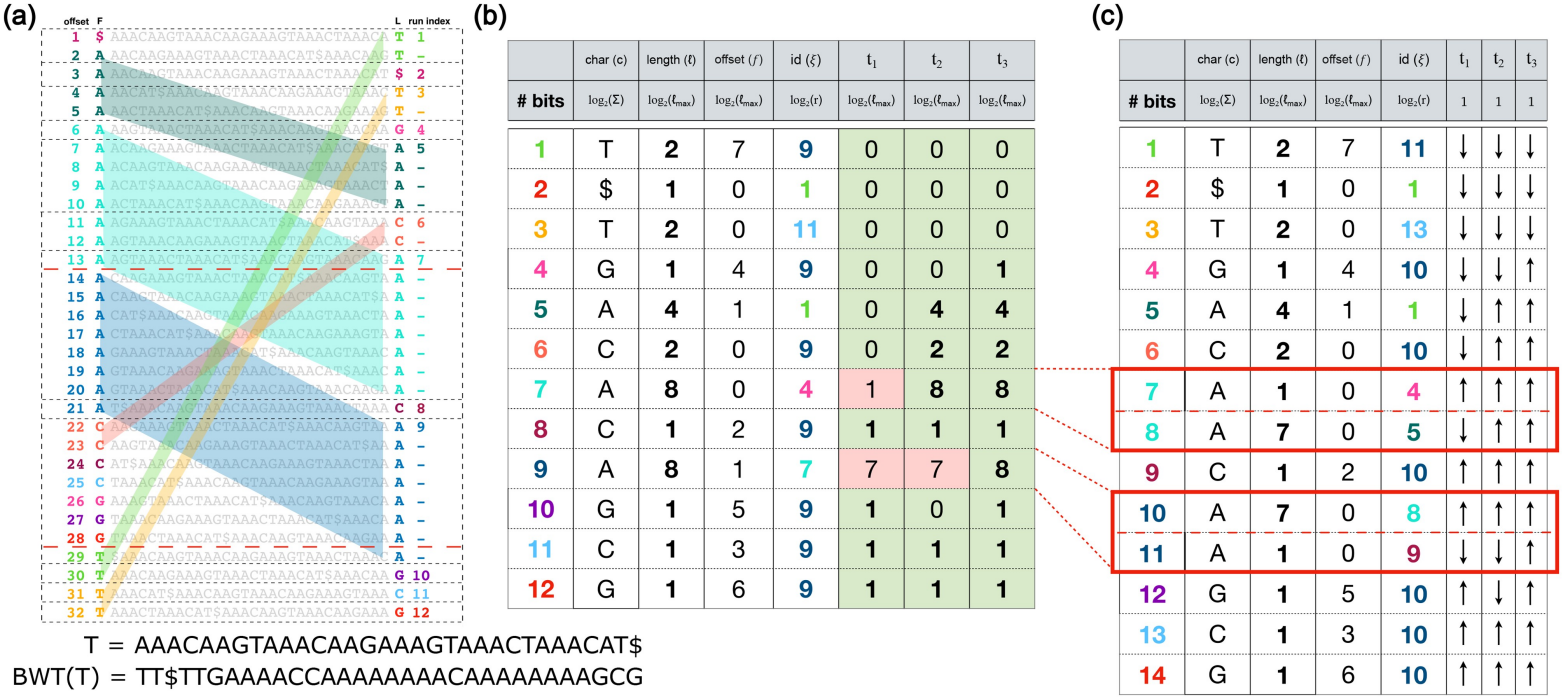
(a) The Burrows Wheeler transform and the Burrows Wheeler matrix built for the sequence T. (b) The Movi index for the sequence T. Trvial thresholds are either 0 or equal to the length of the run.(c) The Movi table after applying splitting based on the non-trivial thresholds. All the thresholds become trivial, allowing them to be represented with a single up/down bit.

The Burrows Wheeler Matrix (BWM) is a sorted array of *T*’s distinct cyclic rotations. The BWM’s last column is BWT(T), as shown in Fig. 1a. The horizontal black dotted lines in Fig. 1a delineate the BWT’s run boundaries. Let *run[i]* denote the *i*th run in the BWT, and let *run[i]*.*h* and *run[i]*.*t* denote the BWT offsets of the first and last letters (respectively) making up the *i*th run. We also call these the run “head” and “tail.” For example, *B[*7*]* is the head of *run[*5*]* in Fig. 1a.

#### 2.1.2 Thresholds

As detailed later, the algorithm that computes pseudo matching lengths includes repositioning steps wherein we might move several rows either up or down in the BWM. Whether we prefer to move up versus down depends on which maximizes the minimum LCP of the BWM rows traversed along the way. The direction that maximizes this LCP also maximizes the length of the match we will later extend, consistent with our goal of computing maximal matches. Rather than explicitly storing or computing these LCPs and their minima, we instead adopt the approach of MONI (Rossi *et al.* 2022), which uses pre-computed values called *thresholds*. For strings $T_{1}$ and $T_{2}$, the longest Common Prefix (LCP) is the length of the longest string *P* that is a prefix of both $T_{1}$ and $T_{2}$. For short, we define LCP(a,b) as the LCP between rows *a* and *b* of the BWM. Let *run[i]* and *run[j]* be two runs of the same character (run[i].c=run[j].c) with i<j such that no intervening run has the same character, i.e. for every *k* with i<k<j, run[k].c≠run[i].c. The threshold $t_{i,j}$ is a BWT offset between *run[i]*.*t* and *run[j]*.*h* which satisfies these conditions:

1. For all *k* in (run[i].t, $t_{i,j}$),LCP(k, run[i].t)≥LCP(k, run[j].h).
2. For all *k* in [$t_{i,j}$, *run[j]*.*h*),LCP(k, run[i].t)≤LCP(k, run[j].h).

Any BWM row offset in [i,j] that is less than this threshold has a greater or equal LCP with the tail of *run[i]* compared to the head of *run[j]*, and any offset greater than or equal to the threshold has a greater or equal LCP with the head of *run[j]* compared to the tail of *run[i]* (Bannai *et al.* 2020). There might be several candidates for the threshold between runs *i* and *j* and any one might be selected. As there are *r* pairs of same-character runs with no intervening runs of the same character, all of the thresholds (one per pair) can be stored in an array of *r* integers.

#### 2.1.3 LF-mapping

The LF-mapping property states that the *i*th occurrence of a character *c* in the last column of the BWM corresponds to the same position in the original text as the *i*th occurrence of *c* in the first column (Ferragina and Manzini 2005). The colored parallelograms in Fig. 1a highlight some of these LF-mapping relationships. For example, LF[5] = 32, because both L[32] and F[5] correspond to position 20 in the text T. Formally, the LF-mapping function, LF(i)=j, maps the BWT offset i to the offset j where *L[i]* and *F[j]* are from the same position *k* of the text *T[k]*. If *F[j]* corresponds to *T[k]*, *L[j]* or BWT[j] corresponds to T[k−1]. Thus, the LF-mapping allows for right-to-left movements with respect to *T*, which is leveraged in pattern-matching queries. The LF-mapping function can be computed as:


$$LF(i)=C(L[i])+\mathrm{ran}k_{L[i]}(i,L),$$

where C(c) is the number of characters in *T* that are lexicographically smaller than *c*, and $\mathrm{ran}k_{c}(i,L)$ is the number of occurrences of character *C* in *L[*1..*i]* (rank of *c* at position *i* of *L*). For example in Fig. 1a, LF(32) = 28 because there are 25 characters (20 *A*s, 4 *C*s, and 1 $) in the text that are lexicographically smaller than *G* and the rank of *G* at offset 32 of the L column (or BWT) is 3.

#### 2.1.4 The move structure

The move structure (Nishimoto and Tabei 2021) is a compressed full-text index based on the run-length encoded BWT of the text. The size of move structure scales by the number of runs (*r*) in the BWT. At the same time, executing an LF-mapping movement takes O(1) time. Also of note, the move structure itself consists of a single table. This is in contrast to other compressed full-text indexes like the r-index, which have many components (e.g. bitvectors or wavelet trees), many of which must be queried at each step of the matching process. Because of this difference, queries to the move structure have better locality-of-reference and are substantially faster Zakeri *et al.* (2024).

There are five columns for each row in a basic move structure: *c* is the character of the run, ℓ is the length of the run, *p* is the BWT offset of the run head, π is the LF-mapping of the run head, and *id* (ξ) is the index of the move row (run) that contains BWT offset π. The parallelograms in Fig. 1a illustrate that the LF-mapping of a BWT offset can be computed based on the distance of BWT offset from its run head and the LF-mapping of the run head. If *M* is the move structure table, and BWT offset i belongs to run index j:


LF(i)=M[j].π+(i−M[j].p).

To determine which run index contains LF(i), we first check whether (j−M[j].π)<M[M[j].ξ].ℓ. If so, then M[j].ξ is the correct next run index. If not, we must scan forward from run M[j].ξ run-by-run until we reach the run containing offset LF(i). We previously called this “fast-forwarding” (Zakeri *et al.* 2024). Nishimoto and Tabei (2021) introduced a row-splitting (or “balancing”) strategy that guarantees a constant number of fast-forward steps while maintaining O(r) runs. Splitting a row with initial length ℓ creates two new rows with lengths $\ell_{1}$ and $\ell_{2}$ such that $\ell_{1}+\ell_{2}=\ell$. While the smaller rows still correspond to equal-letter “runs,” they are no longer maximal. Hence, we use the term “row” to refer generically to both the original rows/runs, and the smaller rows obtained via splitting.

#### 2.1.5 The Movi index

The Movi index uses a move structure table with *r* rows, prior to any splitting. In the following, we will use “run” to refer to BWT runs which are maximal and “move row” or “row” to refer to rows of the move structure. Note that a move row will often correspond exactly to a BWT run, but can also correspond to a partition of a run, due to splitting. Furthermore, a “row-head” refers to the first BWT offset in a move row.

Three of Movi’s columns are identical to those of the move structure (*c*, ℓ, ξ, as seen in Fig. 1b). Unlike the move structure which has columns *p* and π, Movi stores only an offset *f*, the distance of π from the head of $\xi^{\text{th}}$ move row. For example, for move row 9 in Fig. 1a and b, the LF-mapping of the row-head is at BWT offset 14, which is the second BWT offset in move row 7. Its distance from the row-head is 1, so M[9].f=1. The strategy for replacing *p* and π with *f* was suggested by Brown *et al.* (2022).

In Movi, a BWT offset is encoded as a pair (u,v), where *u* is the index of a move row and *v* is the distance from the head of move row *u*. For example, the last BWT offset is encoded as (r,M[r].ℓ−1). This enables navigation through the text without computing full BWT offsets. The LF-mapping function in Movi receives a pair (u,v) as input and outputs $\left(u^{\prime },v^{\prime }\right)$ representing the BWT offset for the LF-mapping of (u,v). This is computed by first computing an intermediate value, $v^{\prime \prime }$, the distance of LF-mapping result from the head of move row M[u].ξ:


$$v^{\prime \prime }=M[u].f+v.$$

The fast-forwarding procedure determines the move row containing this BWT offset and $\left(u^{\prime },v^{\prime }\right)$ is updated according to the cumulative length of the rows skipped during fast-forwarding.

#### 2.1.6 Pseudo matching length queries

Pseudo Matching Lengths (PMLs) are an approximation of matching statistics (MSs). MSs form an array of length m=|P| with entry *MS[i]* equal to the length of the longest prefix of the suffix *P[i*.*m]* that matches a substring of T. PMLs relax the requirement that *PML[i]* equals the maximal prefix of suffix *P[i*.*m]* that matches T; rather, PMLs are usually proportional to but less than the MSs. In return for the approximate answer, the algorithm for computing PMLs is faster than those for computing MSs, as shown by Ahmed *et al.* (2021). Further, PMLs provide similar accuracy in sequence classification tasks compared to MSs (Ahmed *et al.* 2021).

Computing PMLs starts by comparing the rightmost character of the pattern (*P[m]*) to the character at the final offset of the BWT, encoded as (r,M[r].l−1). This process can begin at any offset and we choose the final offset arbitrarily. The process continues by performing iterative LF-mapping steps, interleaved with some repositioning steps using the thresholds. The first PML is set to zero, PML[m]=0, then for every k<m and the BWT offset (i,j):


$$\mathrm{PML}[k]=\{\begin{array}{cc} \mathrm{PML}[k+1]+1 & \text{if}\;P[k]=M[i].c\\ 0 & \text{otherwise}. \end{array}$$

After each *PML[k]* computation, in the case of a match (case 1) the BWT offset is updated by performing LF-mapping. In the case of a mismatch (case 2), we reposition from the current BWT offset to a new offset $\left(i^{\prime },j^{\prime }\right)$ such that $M[i^{\prime }].c=P[k]$, then perform the LF-mapping. Since we always position to a row with a matching character, the LF-mapping following the repositioning is guaranteed to succeed.

For repositioning, Movi scans either up from or down from *i*, moving row-by-row until reaching a row with character matching *P[k]*. Whether the upward or downward candidate row is chosen depends on which direction maximizes the minimum LCP of the rotations traversed along the way. Rather than compute these LCPs and their minima, Movi follows the algorithm of MONI (Rossi *et al.* 2022), which compares *j* with a precomputed “threshold” value (Bannai *et al.* 2020) corresponding to *P[k]* in *M[i]*. If $j<M[i].t_{P[k]}$ then we reposition upward, otherwise downward. Movi must store σ−1 thresholds in a row since a mismatch could involve any character $c^{\prime }\in \Sigma$ such that $c^{\prime }\neq M[i].c$. Note that Movi stores thresholds as an offset in the move row between 0 and *M[i]*.*l* instead of a global BWT offset.

### 2.2 Space efficient indexing with Movi 2

In the default mode of Movi 1, a row of the move table required 16 bytes to store. For Movi 2, we sought to substantially reduce the space required to represent a single row. Figure 1b shows the theoretical space required to store each field in a single row. Storing the character (*c*) requires ${\;\text{log}\;}_{2}(|\Sigma |)$ bits, and the *id* (ξ) requires ${\;\text{log}\;}_{2}(r)$ bits. The length (ℓ), offset (*f*) and threshold values ($t_{1}$, $t_{2}$, $t_{3}$) all require ${\;\text{log}\;}_{2}(\ell_{\text{max}}+1)$ bits, since these are bounded by the maximum length of any move row ($\ell_{\text{max}}$). Movi is designed to support indexing and querying DNA alphabets with alphabet size 4 and therefore stores exactly 3 thresholds per row.

The following sections discuss specific compression techniques, which we also summarize in Section 4, available as supplementary data at *Bioinformatics* online.

#### 2.2.1 Splitting by limiting move row length

Some move-structure columns ($\ell ,f,t_{1},t_{2},t_{3}$) require ${\text{log}\;}_{2}(\ell_{\text{max}}+1)$ bits each to store, since their values can range from 0 to $\ell_{\text{max}}$. To reduce their size, we set an artificial cap on the length of a move row; i.e. we simply set $\ell_{\text{max}}$ according to the number of bits we wish to allocate to these columns, even if some runs exceed that length. We achieve this by splitting any row with length $\ell >\ell_{\text{max}}$ into $\left\lceil \ell_{\text{max}}/\ell \right\rceil$ sub-runs, with all but the last having length $\ell_{\text{max}}$ and the last having length equal to the remainder of $\ell /\ell_{\text{max}}$.

#### 2.2.2 Splitting to compress thresholds

In many cases the threshold value for a particular alphabet character, such as $t_{c}$, is “trivial” in the sense that a mismatch at any of the move row’s offsets should reposition up to the tail of the previous move row with character *c*, or all should reposition down to the head of the next move row with *c*. In this case, the threshold belongs either at the very beginning (offset 0) or the very end (offset ℓ) of the move row. For example, in Fig. 1b, many threshold values are set either to 0 or to ℓ, indicated by the green background. Only three threshold values are neither 0 nor ℓ, indicated by the red background. We call these “non-trivial” thresholds. In Fig. 1a, the red dotted lines highlight the placement of the non-trivial thresholds in the BWM.

If we could eliminate all non-trivial thresholds, a single bit would be sufficient, since it would only need to distinguish whether threshold was at the row’s beginning or end. To achieve this, we propose a new splitting strategy. The goal is to further split the rows of the move structure such that thresholds that were originally non-trivial (i.e. in the middle of a row), such as those denoted with red dotted lines in Fig. 1a, become trivial (i.e. occurring at the move row’s beginning or end). An example of the Movi table before, and after splitting at the thresholds boundaries is shown in Fig. 1b and c. In this example, row 7 with length 8 from Fig. 1b is split into two rows in Fig. 1c: row 7 with length 1 and row 8 with length 1. Similarly, row 9 from Fig. 1b is split into rows 10 and 11 in Fig. 1c. Whereas the original rows 7 and 9 each had at least one non-trivial threshold, the final rows 7, 8, 10 and 11 each have only trivial thresholds.

The splitting will increase the total number of move rows; however, since there are at most *r* thresholds, at most *r* new move rows would be needed, maintaining an overall size bound of O(r). In practice, we observe that splitting the rows at thresholds boundaries increases the number of move rows by only ∼10% of the original number of rows (Table 1). Furthermore, our empirical results demonstrate that the overall effect of thresholds-splitting is a reduction in index size. The thresholds-splitting approach simplifies threshold representation for each character to a binary value, indicating whether the threshold is at the beginning or end of a move row. As a result, storing the thresholds in a Movi index on the DNA alphabet requires 3 bits per move row. This is >16 times smaller than the size of the thresholds in Movi 1.

**Table 1:** Comparing different characteristics of Movi 2 indexes to Movi 1.^a^

Index Type	Bytes per move row	Reference	Additional move rows			Additional structure		Max RSS $ GB)	Index size (GB)	Cache misses (per base).		Bits per BWT run on disk
			Thresholds-splitting.	Length-splitting.	Maximum run length	Relative to move table	Size $ GB)			L1	Last level	
Movi 1	16	Bacteria			$2^{16}-1$	0	0	8.85	8.44	1.85	1.55	160.00^b^
		HPRC Release 1				3.73e-5%	32e-6	82.75	78.92	N/A	N/A	160.00^b^
Movi 2 (default)	8	Bacteria	8.92%	1.60%	$2^{11}-1$			3.92	3.73	1.67	1.21	70.74
		HPRC Release 1	10.49%	0.40%				36.71	35.01	1.81	1.28	70.97
		HPRC Release 2	11.24%	4.63%				47.30	45.11	2.21	1.29	73.86
Movi 2 (Blocked)	6	Bacteria	8.92%	6.73%	$2^{10}-1$	25.47e-5%	8e-6	3.08	2.93	1.98	1.22	55.51
		HPRC Release 1	10.49%	1.34%		25.44e-5%	72e-6	27.77	26.48	2.65	1.25	53.68
		HPRC Release 2	11.24%	13.40%		50.86e-5%	199e-6	38.31	36.54	3.33	1.27	59.82
Movi 2 (Sampled ξ—50)	3	Bacteria	8.92%	19.81%	$2^{9}-1$	13.33%	0.23	1.95	1.85	3.85	3.31	35.02
		HPRC Release 1	10.49%	4.07%			1.94	16.12	15.37	3.87	3.24	31.16
		HPRC Release 2	11.24%	40.60%			3.19	26.45	25.23	4.45	3.29	41.30
Movi 2 (Sampled ξ—100)	3	Bacteria	8.92%	19.81%	$2^{9}-1$	6.67%	0.12	1.83	1.74	5.06	4.46	32.96
		HPRC Release 1	10.49%	4.07%			0.97	15.17	14.47	5.11	4.40	29.33
		HPRC Release 2	11.24%	40.60%			1.59	24.90	23.74	5.67	4.46	38.87
Movi 2 (Sampled ξ—200)	3	Bacteria	8.92%	19.81%	$2^{9}-1$	3.33%	0.06	1.78	1.69	7.60	6.83	31.93
		HPRC Release 1	10.49%	4.07%			0.49	14.70	14.02	7.63	6.78	28.41
		HPRC Release 2	11.24%	40.60%			0.80	24.12	23.00	8.20	6.86	37.66

a Indexes are built on three datasets: Bacteria, HPRC Release 1, and HPRC Release 2. The effect of thresholds and length splitting, the size of the additional data structures, and the number of first and last level cache are shown (cache sizes: L1: 32 KB, LL: 36 608 KB). Movi 1 did not complete successfully on HPRC Release 2 due to high memory usage during index construction.

b The Movi 1 index size is larger than the theoretical expectation based on 16-byte row size, because of padding introduced by memory alignment.

In a later version of Movi 1 (1.2.0), another compression approach (discussed in the Supplementary Materials) based on the trivial and non-trivial thresholds was implemented. That approach reduced the size of each move row to 12 bytes by storing at most 1 non-trivial threshold per move row.

#### 2.2.3 Fewer bits to store for the blocked based design

After applying the compression methods described above, the *id* column (ξ) will typically become the largest component of each move row, requiring ⌈ log(r)⌉ bits to store. By default, Movi 2 uses 36 bits to store ξ, which accounts for more than half the size of a move row. As noted previously (Brown *et al.* 2022), an opportunity to compress ξ comes from the fact that, for rows labeled by the same character, the ξ field is non-decreasing. In fact, for any symbol *c*, ξ can be computed by a rank query in a bit vector of size r that has a set bit at the indices corresponding to the move rows with the symbol *c*. This follows from the LF-mapping, which stably sorts symbols, ensuring that the ξ of the runs are non-decreasing.

Given the ξ values for all the move rows with the same symbol, one approach could be using a piecewise linear function to estimate ξ for move row *i* ($\xi^{\prime }=\text{func}(i,M[i].c)$) and only store the difference ($\Delta =|\xi -\xi^{\prime }|$) at the move row. The difference is a smaller value that requires fewer number of bits for storage. This piecewise-linear fitting strategy is inspired by prior work on learned indexing such as the PGM-Index (Ferragina and Vinciguerra 2020), PLA-index (Abrar and Medvedev 2024), and Sapling (Kirsche *et al.* 2021).

In the blocked mode of Movi 2, we divide the move table into equal size blocks of size *b*. Then, we use a step function with fixed length intervals (length *b*) to estimate ξ for the move rows in each block. For each symbol, the step function returns the ξ of the first move row with that symbol in the block. These values are also called “checkpoints”. The difference (Δ) from the checkpoints are stored in the move row instead of the absolute value of ξ. An example is shown in the Fig. 1, available as supplementary data at *Bioinformatics* online where *b* is set to 8. The Δ column is stored instead of the ξ column in the move rows.

The ξ at the checkpoints are stored in a separate table called the *B* table. For a checkpoint at $i^{th}$ move row, we store |Σ| different ξ values, one for each symbol in the alphabet. The stored ξ for a symbol *y* corresponds to the ξ of a move row with the smallest index $i^{\prime }$ such that $i^{\prime }\geq i$ and $M[i^{\prime }].c=y$ (i.e. $i^{\prime }$ is the first occurrence of *y* at or after *i* in the block).

If we choose *b* large enough, the number of blocks is reduced such that the *B* table fits into cache. That makes the retrieval of checkpoints very fast. In practice, we see that the number of last level cache misses never increases by >1%, and in some cases, even decreases. For the first level cache, depending on the size of the *B* table, cache misses increase by 19%–51%. Overall, this approach achieves space efficiency without noticeably compromising speed.

We can use the $\Delta_{\xi }$ and the B table to recompute the original ξ values for a row *i* with character *c*. The closest checkpoint before *i* is computed as $i^{\prime }=i/b$, then the ξ is computed as:


$$\xi =B[i^{\prime }][c]+M[i].\Delta_{\xi }$$

#### 2.2.4 Sampling the ξ field at the checkpoints

We can extend the blocking idea to a sampling approach which does not store Δ values explicitly. Instead, the Δ may be inferred by computing the distance of a row-head from a checkpoint, i.e. head of a sampled move row, by scanning the move rows in between. We denote the sampling rate by s and use the same method as in the blocked design to store ξ for each symbol at the checkpoints. In this mode, the table containing the ξs of sampled move rows is referred to as the S table.

To find the ξ for a move row *q* with symbol *y*, we examine move rows between *q* and the closest sampled row. Assume the closest sampled move row is at index i<q. First, we must scan the move rows [i,q) and compute the cumulative length of rows having symbol *y*. This will be the distance in terms of BWT offsets between heads of move rows *i* and *q* after LF-mapping. The distance after the LF-mapping is important, because the ξ of move row *q* is the index of the move row containing the LF-mapping of the head of move row *q*. We can now compute the ξ of move row *q*, given the ξ for symbol *y* at sampled move row *i* and the corresponding offset for that. The offset of the sampled move rows can be retrieved by simply examining the content of the move row, since *f* (the offset) is stored for all rows.

**Figure 2:**
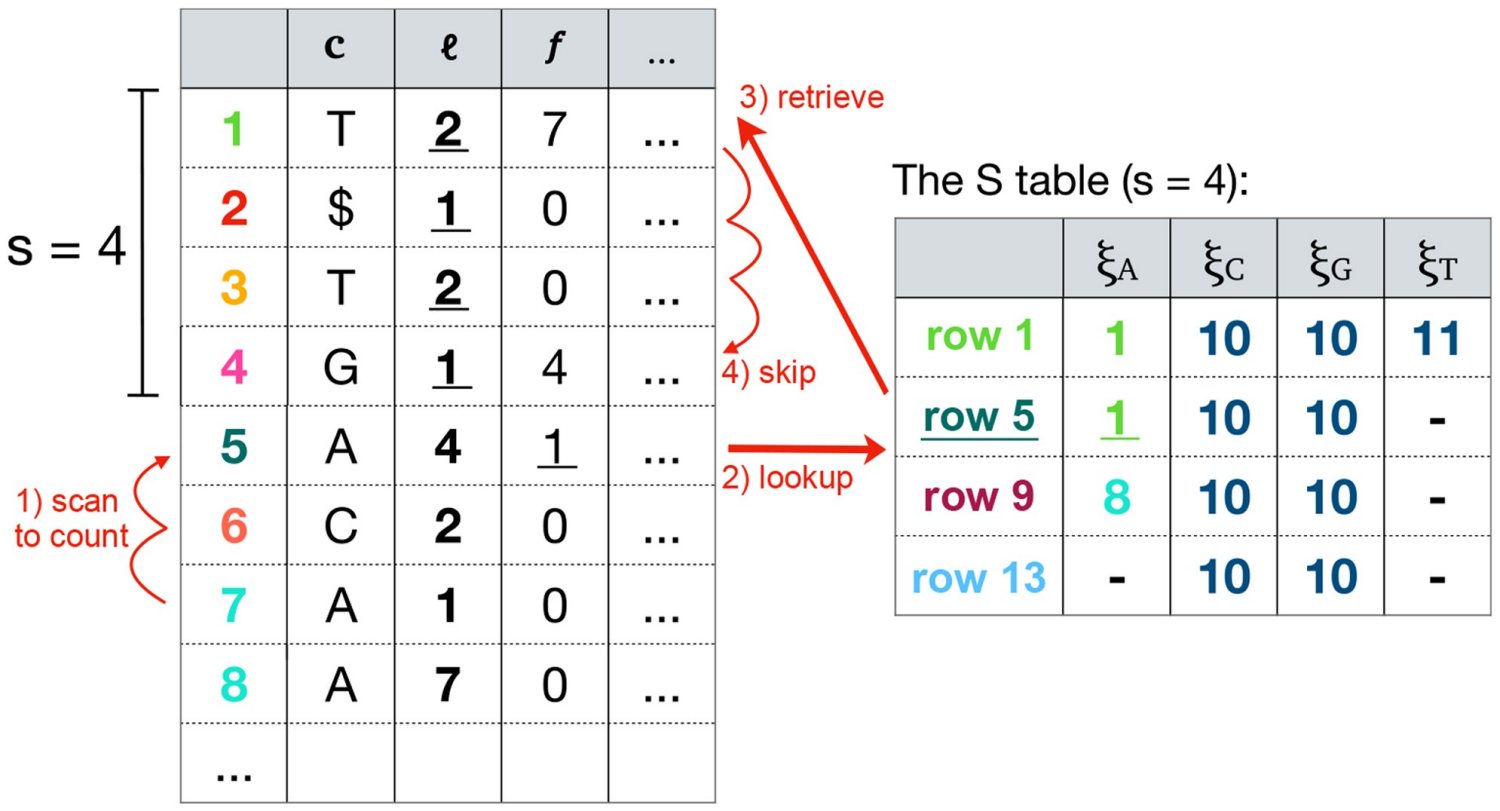
The sampled design where no *id* (ξ) is stored in the move rows. Instead, ξ at sampled move rows are stored in the S table. As an example, the ξ of move row 7 is computed in four steps: (1) scan to count the 4 As between the move rows 7 and sampled row 5, (2) lookup the ξ for A at row 5 which is 1, (3) retrieve the move row identified by the ξ (row 1), and (4) scan move rows until 4 BWT offsets (as many as the number of As counted in step 1) are skipped from the BWT offset (1, 1). The final move row reached (row 4) is the ξ of move row 7.

The process at this point becomes similar to a fast-forward step. Given the LF-mapping of a BWT offset, we want to compute the LF-mapping of another BWT offset at a certain distance. This step invokes another scan from the retrieved ξ for *y* until the desired BWT offset is reached. We illustrate this procedure through an example in Fig. 2 where we compute the ξ of row 7 based on the sampled move rows. A pseudo code of this process is also provided in Algorithm 1, available as supplementary data at *Bioinformatics* online.

#### 2.2.5 Summary of compression variants

Section 4, available as supplementary data at *Bioinformatics* online summarizes the relationship between the compression methods discussed above, the core idea behind each, and the qualitative trade-offs between space usage and query speed. The table also highlights how combinations of compression techniques become “modes” of the tool, where the user can select a mode depending on whether memory footprint or query performance is the primary constraint.

## 3 Results

### 3.1 Analyzing different versions of Movi 2

To compare the Movi 2 variants to Movi 1, we used three different datasets: (i) a collection of 7692 complete genomes from 7 bacterial species (“Bacteria”), (ii) 94 human haplotypes from the first release of Human Pangenome Reference Consortium (“HPRC Release 1”) (Liao *et al.* 2023), and (iii) 466 human haplotypes, consisting of 464 haplotypes from the second HPRC release (May 12, 2025) plus CHM13 (Nurk *et al.* 2022) and GRCh38 (Schneider *et al.* 2017) (“HPRC Release 2”). More details about total lengths and number of BWT runs for each reference is provided in Table 3, available as supplementary data at *Bioinformatics* online.

First, we compared different versions of the Movi 2 index with Movi 1 and evaluated how Movi 2 compression strategies affected different aspects of the index. Movi 1 uses 16 bytes per row, while the default Movi 2 uses 8 bytes. The blocked design uses 6 bytes and the smallest version (sampled-ξ) uses 3 bytes per row. The sampled-ξ version provides speed-memory trade-off by varying the sampling rate. Here we tried 50, 100, and 200.

The smaller row size in default Movi 2 is achieved via two row-splitting strategies. The first, thresholds-splitting, forces all the thresholds to be trivial, allowing a single threshold status bit to be stored instead of a full threshold value. The second, length splitting, reduces the bits required to store length and offset columns. As there are at most r distinct thresholds, in the worst case, the thresholds-splitting doubles the number of rows. However, in practice we observed far fewer rows are added. Table 1 shows that thresholds splitting increased the number of move structure rows by 8.92% to 11.24% in different datasets.

Table 1 also shows the percentage increase in rows due to length-splitting. In default Movi 2, we use 11 bits for the length (ℓ) and offset (*f*) columns. For the blocked index, we use 10, and sampled-ξ uses 9 bits. When fewer bits are used, more splitting is required. With maximum run length of $2^{11}-1$, the number of rows was increased by 1.60%, 0.40%, and 4.63% for the Bacteria, HPRC Release 1, and HPRC Release 2 indexes, respectively. For the blocked indexes, the increase in the number of rows was between 1.34% and 13.4%. The largest growth in our experiments was observed for the sampled-ξ variants, where the number of rows was increased between 4.07% and 40.60%.

For length-splitting, we observed that the amount of splitting required was dataset dependent. Highly repetitive datasets will also have high n/r and so (by definition) longer average run lengths. The Bacteria dataset spans many species but includes some collections of thousands of genomes from the same species with n/r (average run length) of 157.94. The HPRC Release 1 dataset, consists of 94 human haplotypes and has an n/r of 133.80 while the HPRC Release 2 includes 466 human haplotypes with n/r of 535.02. For HPRC Release 2 dataset, we saw an increase of 40.60% in the number of move structure rows when the maximum run length is bounded by $2^{9}-1$. However, since the sampled-ξ version only uses 3 bytes per row (>80% less than the original Movi index), the overall size of the index becomes smaller.

The “Additional structure” column in Table 1 shows the absolute and relative size of the additional table used in some indexes compared to the main move structure table. For Movi 1, this additional table stores rare move rows that do not fit in the main table because their length, offset, or threshold values require >16 bits to represent. For the blocked and sampled-ξ indexes, this column shows the size of the B table and S table, respectively. The size of the B table depends on the number of checkpoints and the size of the blocks. It was small in our experiments: 8 KB for Bacteria, 72 KB for HPRC Release 1, and 199 KB for HPRC Release 2. For HPRC Release 2, we had to select a smaller block size to guarantee all deltas could fit in the $\Delta_{\xi }$ column. For the sampled-ξ version, we evaluated sampling rates of 50, 100, and 200, which result in additional tables that are 13.33%, 6.67%, and 3.33% of the size of the main table, respectively. Total index size and (including additional data structures) and the max RSS during runtime is also shown in Table 1. Furthermore, the last column of Table 1 reports the average number of bits per BWT run on disk, computed by dividing the total index size by the original number of runs in the BWT (before any splitting).

To assess how the compression strategies affected locality-of-reference, we used the “Cachegrind” profiler to measure cache misses at the first (L1) and last (LL) cache levels. Default Movi 2 had lower L1 and LL (last level) cache misses due to its smaller size compared to Movi 1. For the variants with extra table (blocked and sampled-ξ), the number of cache misses increased due to extra accesses to either the B or S tables. The B table is small enough to fit in the last-level cache of most systems. This was evident as the number of LL cache misses was not significantly increased, and even decreased in some cases due to smaller size of the main table. For the sampled-ξ index, the number of cache misses increased with higher sampling rates because more scanning in the main table was needed to get to a sampled move row.

### 3.2 Movi 2’s space/speed trade-off

We performed benchmarks to assess the space/time trades achievable by Movi 2’s compression methods. We used long reads to compare Movi 2’s PML query to the same query performed by Movi 1 and SPUMONI (which uses the *r*-index). We also included ropebwt3 (Li 2024) in our benchmark which is another scalable tool for full-text indexing of pangenomes, though it supports a different and broader set of queries compared to the other tools in the benchmark. In this experiment, ropebwt3 was used to compute Super Maximal Exact Matches (SMEMs). We used ropebwt3’s static index format, called FMD, since this achieves lower memory footprint and higher speed compared to the FMR dynamic index format. Also for ropebwt3, we ran three experiments; one with a SMEMs minimum length threshold of 19, one with a minimum SMEM threshold of 31, and a third with a minimum SMEM threshold of 51. These experiments were meant to cover a range of time/space tradeoffs for ropebwt3. All the tools (Movi, Movi 2, SPUMONI, and ropebwt3) include both strands of the reference sequences (forward and reverse complement) in the index, since this facilitates both bi-directional matching and the identification of matches on both strands with a single query.

For the bacteria dataset, we used 100K ONT reads from SRR11071395, the Zymo High Molecular Weight Mock Microbial Community (Kovaka *et al.* 2021). These had an average length of 15K bases. For the HPRC datasets we used PBSIM2 (Ono *et al.* 2021) to simulate 1.18 M reads from the CHM13 with an average length of 2.6K bases.

**Figure 3:**
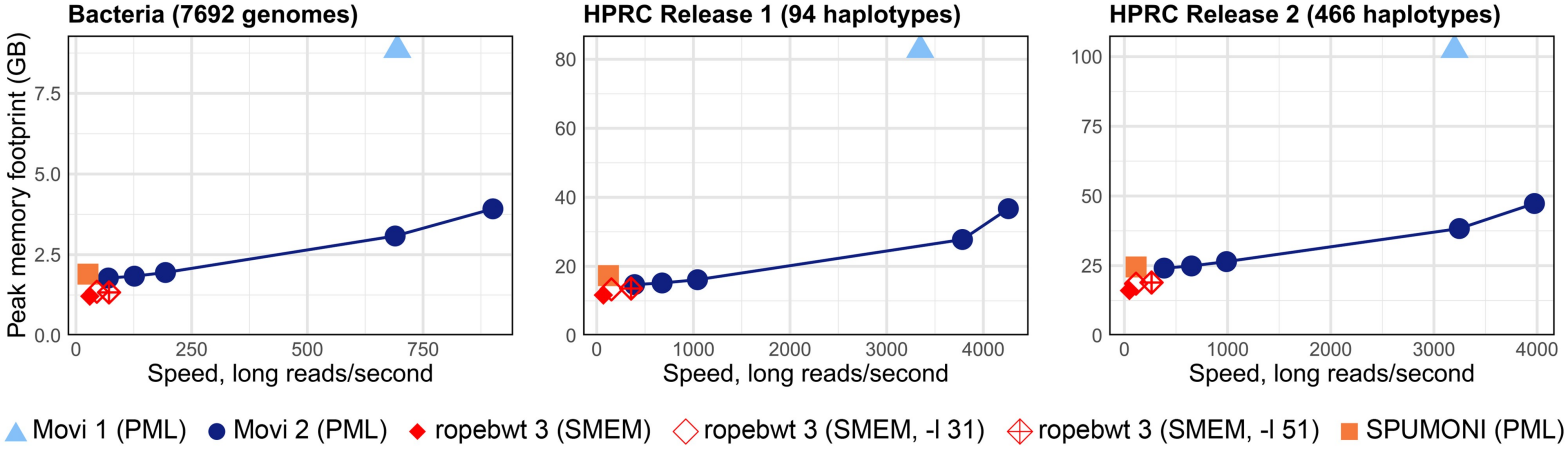
The time and maximum resident set size (max RSS) for querying different indexes. Movi, Movi 2, and SPUMONI compute PMLs. ropebwt3 computes Super Maximal Exact Matches (SMEMs) of length at least 19 (solid diamond), at least 31 (open diamond), or at least 51 (diamond with cross). All the tools are run with 1 thread and the average of five consecutive runs for each method is reported. Movi benefits from latency hiding by default (Zakeri *et al*. 2024). The 5 points along the Movi 2 line represent, from right to left, the threshold-split, blocked, sampled-50, sampled-100 and sampled-200 modes, where the number after “sampled” indicates the interval between samples. The bacteria indexes are built on 7692 genomes from seven different bacterial species, then queried by 100K long ONT reads (Zymo). For HPRC experiments 1 188 163 long reads simulated from the CHM13 reference are used for the query. We profiled the static (index file ending in .fmd) variants of the ropebwt3 index, since these are generally smaller and faster to query compared to the dynamic (.fmr index files). Please refer to Table 4, available as supplementary data at *Bioinformatics* online for more details. *Note that Movi 1 result on HPRC Release 2 is estimated as the run did not complete successfully due to high memory usage during index construction.

Figure 3 presents the results, with five points corresponding to the different compression/speed trade-offs implemented in Movi 2, alongside the results from Movi 1, SPUMONI, and ropebwt3. More details about the speed can also be found in Table 4, available as supplementary data at *Bioinformatics* online. The fastest method was default Movi 2 which also had a lower peak memory footprint compared to Movi 1. PML query with this mode was ∼35 times faster than the same query performed by SPUMONI. The default Movi 2’s index was 56% smaller than Movi 1 in all three different datasets. The PML query with this Movi 2 index was also between 27% and 30% faster than Movi 1, likely due to its decreased rate of cache misses and smaller index.

The next fastest mode of Movi 2 is the blocked mode which was between 19% and 24% smaller than default Movi 2. This mode required 65% less memory compared to Movi 1. Query with the blocked Movi 2 was ∼13% faster than Movi 1 for the HPRC Release 1 dataset. For the bacteria dataset, the speed of blocked mode query was <1% slower than Movi 1.

The next three points for Movi 2 correspond to sampled-ξ indexes with sampling rates of 50, 100, and 200. Higher sampling rates resulted in smaller index and slower query. Using different sampling rates, Movi 2 was able to use less memory than SPUMONI in all three different datasets while remaining faster. In the Bacteria dataset, Movi 2’s memory usage became smaller than SPUMONI at sampling rate of 100 while being 4.86x faster. In HPRC Release 1, the sampled-ξ Movi 2 was smaller than SPUMONI but 20%–40% larger than ropebwt3 across the configurations we evaluated. For sampling rate of 50, Movi 2’s PML computation was 8.61x faster than SPUMONI ’s PML computation. It was also 15.1×, 6.8×, and 2.9× faster than ropebwt3’s SMEM finding mode using minimum match lengths of 19 (default), 31, and 51, respectively. Though we evaluated three different minimum length thresholds, we emphasize that SMEMs are more difficult results to compute compared to PMLs in general.

The HPRC Release 2 dataset had a high average run length that caused more length splitting in the sampled-ξ mode. For this reason, a higher sampling rate (200) was needed for Movi 2’s index to become smaller than SPUMONI ’s. In that scenario Movi 2 was still 3.38x faster than SPUMONI. Overall, the results demonstrate that indexes based on move structure are able to achieve simultaneous speed/memory improvements compared to *r*-index based methods.

### 3.3 Parallelism and latency hiding

**Figure 4:**
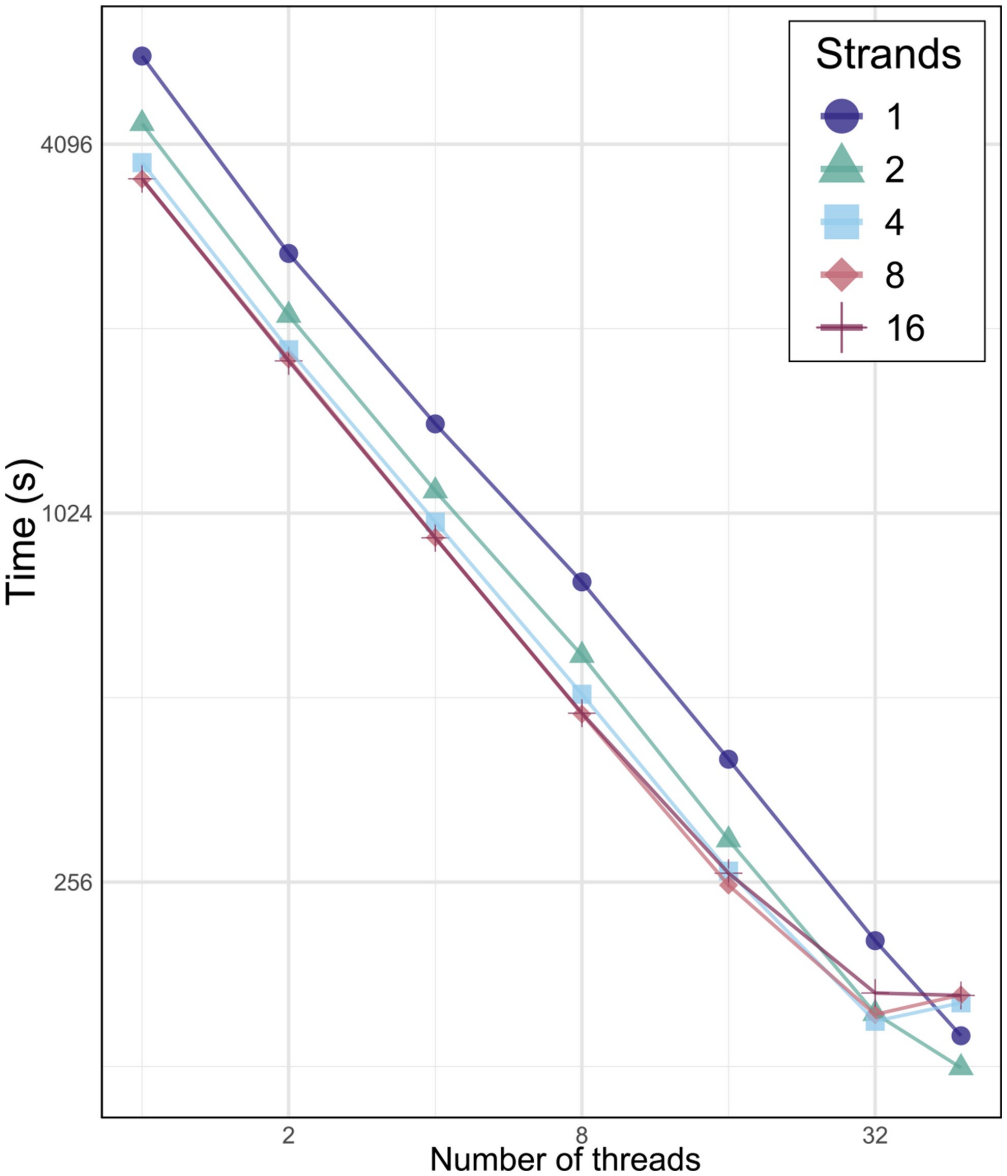
Movi 2’s performance with different number of threads and strands (for latency hiding). The number of concurrent reads processed per thread is referred to as strands.

Latency hiding by prefetching is one of the features enabled in Movi because it is based on the move structure. Movi 1 and Movi 2 are able to concurrently process multiple reads (called strands) with a single thread to achieve latency hiding. The Movi and Movi 2 results in Fig. 3 all use latency hiding, for example. Movi 2 further extends this by offering parallelization across multiple threads, in addition to concurrent read processing within each thread. Figure 4 shows how Movi 2’s performance scaled as the number of threads increased. For each thread count, we also evaluated the effect of prefetching. Our results showed that prefetching improved performance even under thread-level parallelization.

We conducted experiments on a system with 48 hardware threads, scaling up to full utilization. We observed that when all threads are used, performance slows down, likely due to system saturation and competition for compute resources. In this case, prefetching can have an inverse effect, further intensifying resource contention.

## 4 Discussion

As pangenomes grow in size and utility, there is a growing need to improve the size and speed of pangenome indexes. The move structure is a compressed full-text index that has been shown to be drastically faster than others like the *r*-index. While this was thought to inevitably come at the price of a larger data structure, here we showed that the move structure can be both smaller and faster than an *r*-index. We implemented these ideas in the Movi 2 software, and further showed that a range of these ideas yield a flexible trade-off space such that Movi 2 dominates either the *r*-index or the previous version (Movi) when configured appropriately.

A question for future work is whether the move structure, including the ideas proposed here, can be combined with minimizer digestion in order to improve the efficiency of sequence classification. This was shown to be possible using the r-index in the SPUMONI2 study (Ahmed *et al.* 2023). That study also showed that this has computational benefits, including (i) the addition of a layer of lossy compression (by way of minimizer digestion) on top of the lossless compression already achieved by the BWT, (ii) a reduction in the number of iterations required in the matching inner loop, since the loop can proceed minimizer-by-minimizer rather than character-by-character. However, it is not yet known if the move structure can benefit similarly. An obstacle is that minimizer digestion in its most typical form will increase the size of the alphabet, since the alphabet is “promoted” from the A/C/G/T nucleotides to the larger set of possibly minimizer sequences. The SPUMONI2 study uses 4-character minimizers such that the minimizer alphabet has size $4^{4}$ = 256. Increasing the alphabet size introduces a particular challenge for storing thresholds in the current design of Movi 2. Each additional alphabet symbol requires its own threshold, since a new bit must be allocated to represent it. One way to address this is to store thresholds in two groups: one for characters with a threshold bit of 0 and another for those with a threshold bit of 1. We hypothesize that the number of distinct ways that characters will fall into these 0/1 groups will be limited, allowing them to be represented with an integer id that uses <256 bits. A further complication arises in repositioning, as it may now require scanning farther across nearby rows to locate a move row with the appropriate “character” (i.e. minimizer). Further investigation is required to determine if the move structure can maintain its efficiency advantage even for larger alphabets.

A question for future work is how the move structure and Movi 2 will scale to still larger pangenomes, e.g. thousands of human haplotypes. The move structure’s efficiency arises in part from its compact, row-major layout. But this also introduces rigidity: the bit widths of individual fields (e.g. row length, offsets, and ξ) must be fixed in advance. But these trades only fit every dataset. For example, datasets with lower compression ratios (smaller n/r) tend to increase the number of rows and therefore the range of ξ, while repetitive datasets (larger n/r) increase the maximum row length and associated offsets.

Since the optimal allocation of bits across fields can vary substantially depending on the input, fixed-width designs such as those used in Movi 2 may fail to fit some new pangenomes. One possible solution is to develop more flexible representations that adapt field widths to the input dataset at construction time, rather than fixing them a priori. Such approaches could better balance space across components by tailoring bit allocations to the observed distribution of run lengths and ξ values. However, this flexibility may come at a cost: for example, supporting non-uniform or non-byte-aligned field widths could complicate navigation and increase computational overhead. Exploring these trade-offs between flexibility, compression, and query efficiency will be an important direction for scaling move-structure-based indexes to future pangenome collections.

Another direction for future work is to further reduce the space usage of the move table by shrinking some of the fields that have so far received less attention. The offset field *f* for example, might be amenable to some of the same compression ideas as the *id* field.

Also, a small but important remaining issue is the handling of non-A/C/G/T characters in the reference collection, such as Ns. Currently, such letters are converted to randomly selected characters from the set {A, C, G, T}, but in the future it will be important to preserve them and to force them to mismatch other characters as is common in other read alignment tools.

## Supplementary Material

btag362_Supplementary_Data

## Data Availability

The Movi 2 software is available under the open source GPL-3.0 license at https://github.com/mohsenzakeri/Movi. An archival release of the code (version 2.0.0) is available at https://doi.org/10.5281/zenodo.18868297.

## References

[btag362-B1] Abrar MH , Medvedev P. Pla-index: a k-mer index exploiting rank curve linearity. LIPIcs Leibniz Int Proc Inf 2024;312:13:1–13:18. 10.4230/LIPIcs.WABI.2024.13 PMC12037174 40297743

[btag362-B2] Ahmed O , Rossi M, Kovaka S et al Pan-genomic matching statistics for targeted nanopore sequencing. iScience 2021;24:102696. 34195571 10.1016/j.isci.2021.102696 PMC8237286

[btag362-B3] Ahmed OY , Rossi M, Gagie T et al Spumoni 2: improved classification using a pangenome index of minimizer digests. Genome Biol 2023;24:122. 37202771 10.1186/s13059-023-02958-1 PMC10197461

[btag362-B4] Bannai H , Gagie T, Tomohiro I. Refining the r-index. Theor Comput Sci 2020;812:96–108.

[btag362-B6] Brown NK , Gagie T, Rossi M. RLBWT tricks. In: Schulz C, Uçar B (eds), 20th International Symposium on Experimental Algorithms (SEA 2022), Volume 233 of Leibniz International Proceedings in Informatics (LIPIcs). Dagstuhl, Germany: Schloss Dagstuhl – Leibniz-Zentrum für Informatik, 2022, 16:1–16. 10.4230/LIPIcs.SEA.2022.16; https://drops.dagstuhl.de/opus/volltexte/2022/16550

[btag362-B7] Burrows M , Wheeler DJ. A block-sorting lossless data compression algorithm. Digit Equip Corporat, 1994.

[btag362-B8] Chen N-C , Solomon B, Mun T et al Reference flow: reducing reference bias using multiple population genomes. Genome Biol 2021;22:8. 33397413 10.1186/s13059-020-02229-3 PMC7780692

[btag362-B9] Ferragina P , Manzini G. Indexing compressed text. J ACM 2005;52:552–81.

[btag362-B10] Ferragina P , Vinciguerra G. The pgm-index: a fully-dynamic compressed learned index with provable worst-case bounds. Proc VLDB Endowment 2020;13:1162–75.

[btag362-B11] Gagie T , Navarro G, Prezza N. Optimal-time text indexing in BWT-runs bounded space. In: *Proceedings of the Twenty-Ninth Annual ACM-SIAM Symposium on Discrete Algorithms, New Orleans, LA, USA*. Philadelphia, PA, USA: SIAM, 2018, 1459–77.

[btag362-B12] Gagie T , Navarro G, Prezza N. Fully functional suffix trees and optimal text searching in BWT-runs bounded space. J ACM 2020;67:1–54.

[btag362-B13] Kirsche M , Das A, Schatz MC. Sapling: accelerating suffix array queries with learned data models. Bioinformatics 2021;37:744–9. 33107913 10.1093/bioinformatics/btaa911

[btag362-B14] Kociumaka T , Navarro G, Prezza N. Towards a definitive compressibility measure for repetitive sequences. IEEE Trans Inform Theory 2023;69:2074–92.

[btag362-B15] Kovaka S , Fan Y, Ni B et al Targeted nanopore sequencing by real-time mapping of raw electrical signal with uncalled. Nat Biotechnol 2021;39:431–41. 33257863 10.1038/s41587-020-0731-9 PMC8567335

[btag362-B16] Kuhnle A , Mun T, Boucher C et al Efficient construction of a complete index for pan-genomics read alignment. J Comput Biol 2020;27:500–13. 32181684 10.1089/cmb.2019.0309 PMC7185338

[btag362-B17] Li H. Bwt construction and search at the terabase scale. Bioinformatics 2024;40:btae717. 39607778 10.1093/bioinformatics/btae717 PMC11646566

[btag362-B18] Liao W-W , Asri M, Ebler J et al A draft human pangenome reference. Nature 2023;617:312–24. 37165242 10.1038/s41586-023-05896-x PMC10172123

[btag362-B19] Lin M-J , Iyer S, Chen N-C et al Measuring, visualizing, and diagnosing reference bias with biastools. Genome Biol 2024;25:101. 38641647 10.1186/s13059-024-03240-8 PMC11027314

[btag362-B20] MäKinen V , Navarro G. Succinct suffix arrays based on run-length encoding. In: Combinatorial Pattern Matching: 16th Annual Symposium, CPM 2005, Jeju Island, Korea, June 19-22, 2005. Proceedings. Vol. 16. Berlin, Heidelberg: Springer, 2005, 45–56.

[btag362-B21] Mun T , Kuhnle A, Boucher C et al Matching reads to many genomes using r-index. J Comput Biol 2020;27:514–8. 32181686 10.1089/cmb.2019.0316 PMC7185317

[btag362-B22] Nishimoto T , Tabei Y. Optimal-time queries on BWT-runs compressed indexes. In: *48th International Colloquium on Automata, Languages, and Programming (ICALP)*. Vol. 198, Dagstuhl, Germany: Schloss Dagstuhl–Leibniz-Zentrum fur Informatik, 2021, 101.

[btag362-B23] Nurk S , Koren S, Rhie A et al The complete sequence of a human genome. Science 2022;376:44–53. 35357919 10.1126/science.abj6987 PMC9186530

[btag362-B24] Ono Y , Asai K, Hamada M. Pbsim2: a simulator for long-read sequencers with a novel generative model of quality scores. Bioinformatics 2021;37:589–95. 32976553 10.1093/bioinformatics/btaa835 PMC8097687

[btag362-B25] Pritt J , Chen N-C, Langmead B. FORGe: prioritizing variants for graph genomes. Genome Biol 2018;19:220. 30558649 10.1186/s13059-018-1595-x PMC6296055

[btag362-B26] Raskhodnikova S , Ron D, Rubinfeld R et al Sublinear algorithms for approximating string compressibility. Algorithmica 2013;65:685–709.

[btag362-B27] Rossi M , Oliva M, Langmead B et al MONI: a pangenomic index for finding maximal exact matches. J Comput Biol 2022;29:169–87. 35041495 10.1089/cmb.2021.0290 PMC8892979

[btag362-B28] Schneider VA , Graves-Lindsay T, Howe K et al Evaluation of grch38 and de novo haploid genome assemblies demonstrates the enduring quality of the reference assembly. Genome Res 2017;27:849–64. 28396521 10.1101/gr.213611.116 PMC5411779

[btag362-B29] Zakeri M , Brown NK, Ahmed OY et al Movi: a fast and cache-efficient full-text pangenome index. iScience 2024;27:111464. 39758981 10.1016/j.isci.2024.111464 PMC11696632

