# Supplementary material for: Movi 2: fast and space-efficient queries on pangenomes
Source: Bioinformatics. 2026 Jun 17;42(7):btag362. doi: 10.1093/bioinformatics/btag362 (PMC13379629; doi:10.1093/bioinformatics/btag362)
Supplement: btag362_Supplementary_Data [file btag362_supplementary_data.zip › Movi_2_supp.pdf]

Supplementary Materials for Movi 2

1. Storing only the non-trivial thresholds

As an advanced version of Movi 1, we implemented a specific thresholds compression approach to store a threshold status instead of an offset for each character. A threshold-offset for each character requires $O(\log_2(\ell_{max}))$ bits (2 bytes in Movi), whereas a threshold-status requires only 2 bits. This is because the status needs to express three main possibilities (assuming the length of the move row is ℓ):

1) The threshold is at the start of the move row (0).
2) The threshold is at the end of the move row (ℓ).
3) a non-trivial offset inside the move row (between 0 and ℓ).

The first two cases do not require any additional information to retrieve the threshold offset. In case (1), the threshold offset is 0 and in case (2), it is set to the length of the the move row. For case (3), the explicit offset of the non-trivial threshold is stored in a dedicated field which requires $O(\log_2(\ell_{max}))$. However, this is the only column requiring $O(\log_2(\ell_{max}))$ bits, which is an improvement over storing $\Sigma - 1$ columns with the same number of bits. It is also possible, though rare in our experiments, for multiple non-trivial thresholds to exist in a row. This can be identified using the 4th code of the threshold status. To handle such cases, we store an overflow table that explicitly stores the thresholds for rows with more than one non-trivial threshold. In practice, we find that this table is small compared to the main Movi table. This approach was implemented in a later release of Movi 1, reducing the total size of each move row from 16 bytes to 12 bytes.

When a trivial threshold is represented by a threshold status rather than full offset, the most significant part of the threshold data structure in Movi becomes the non-trivial thresholds.

2. The blocked index

b = 8 (rows 1–8)

	c	ℓ	f	Δ_ξ	...
1	T	**2**	7	0	...
2	$	**1**	0	0	...
3	T	**2**	0	2	...
4	G	**1**	4	0	...
5	A	**4**	1	0	...
6	C	**2**	0	0	...
7	A	**1**	0	3	...
8	A	**7**	0	0	...
9	C	**1**	2	0	...
...					

The B table (b = 8):

	ξ_A	ξ_C	ξ_G	ξ_T
row 1	1	10	10	11
row 9	8	10	10	-

Fig. 1: The blocked design of Movi 2 where the exact *id* at checkpoints are stored explicitly for each block in the B table. The block size is 8 in this example. The difference from the checkpoint is stored in the move row.

3. Computing the id in the sampled-id mode

Algorithm 1 Computing the id for move row q using the S table. M is the Move table, s is the sampling rate.

Require: M, q, s, S
$C \leftarrow M[q].c$
if $q \,\%\, s = 0$ **then**
    $\xi \leftarrow S[q/s][C]$ ▷ q is at a checkpoint, simply retrieve the sampled id.
else
    $i \leftarrow \lfloor q/s \rfloor \times s$ ▷ the index of the checkpoint in the move table
    $d \leftarrow 0$
    while $q > i$ **do** ▷ step (1) scan and count
        $i \leftarrow i - 1$
        if $M[q].c = C$ **then**
            $d \leftarrow d + M[q].\ell$
        end if
    end while
    $u \leftarrow \lfloor q/s \rfloor$ ▷ step (2) lookup the index of the sampled row in the S table
    $\xi \leftarrow S[u][C]$ ▷ step (3) retrieve the sampled id
    $\ell' \leftarrow M[\xi].\ell - M[i].f$ ▷ the distance from offset until the end of the row
    $d \leftarrow d - \ell'$ ▷ step (4) skip
    while $M[\xi].\ell < d$ **do**
        $\xi \leftarrow \xi + 1$
        $d \leftarrow d - M[\xi].\ell$
    end while
end if
return ξ

4. Summary of Movi 2 variants

Variant	Bytes per Move row	Builds On	Thresholds	id (ξ)	Use Case
**Movi 1**	16	–	Store all non-trivial thresholds $t_{c'}$	Store full ξ per row	Baseline; superseded by Movi 2
**Movi 2** Thresholds-Split (Default)	8	Movi 1	Split until **all** thresholds are trivial (1 bit each)	Full ξ stored per row	Movi 2 default; strong space reduction (roughly 2-fold) and higher speed compared to Movi 1
**Movi 2** Blocked	6	Thresholds-Split	All trivial (1 bit each)	Store ξ as difference from periodically stored checkpoints	Intermediate memory savings and moderate query slowdown
**Movi 2** Sampled	3	Thresholds-Split	All trivial (1 bit each)	Store ξ checkpoints in separate S-table (not counted in 3 bytes/row); compute ξ via scan between checkpoints	Maximum compression; best for memory-constrained settings

Table 2. Summary of the relationships between the Movi 2 compression variants. Each variant modifies how thresholds and/or destination identifiers (ξ) are stored. Blocked and Sampled are alternative encodings built on the thresholds-split structure. Note that all Movi 2 variants use the "limiting move row length" splitting idea as well.

5. Statistics of input datasets

Dataset	# Genomes or Haplotypes	Total Length incl. RC (n)	# BWT Runs (r)	n/r
Bacteria	7692	71,502,400,381	452,717,159	157.94
HPRC Release 1	94	566,867,856,061	4,236,540,383	133.80
HPRC Release 2	466	2,806,441,715,737	5,245,449,934	535.02

Table 3. Summary statistics of the reference sequences used in the experiments. Note that the actual number of sequences in each index is twice the number of genomes or haplotypes since we also include the reverse complement (RC) of each sequence. The total lengths and number of BWT runs are calculated after including the reverse complements.

6. Speed of different methods on different references

Method	Reference	Reads per Second	Symbols per Second
Movi 1 (PML)	Bacteria	694.44	10,958,904.11
Movi 2 (PML)	Bacteria	900.90	14,216,661.93
Movi 2 (blocked deltas)	Bacteria	689.66	10,883,761.43
Movi 2 (sampled id 50)	Bacteria	193.42	3,052,316.71
Movi 2 (sampled id 100)	Bacteria	126.26	1,992,508.17
Movi 2 (sampled id 200)	Bacteria	69.98	1,104,313.45
SPUMONI (PML)	Bacteria	25.96	409,676.56
ropebwt3 (SMEM)	Bacteria	29.98	473,043.61
ropebwt3 (SMEM, -l 31)	Bacteria	45.19	713,093.83
ropebwt3 (SMEM, -l 51)	Bacteria	71.48	1,127,993.41
Movi 1 (PML)	HPRC Release 1	3,346.94	8,784,258.61
Movi 2 (PML)	HPRC Release 1	4,258.65	11,176,930.81
Movi 2 (blocked deltas)	HPRC Release 1	3,783.96	9,931,472.84
Movi 2 (sampled id 50)	HPRC Release 1	1,042.25	2,735,529.05
Movi 2 (sampled id 100)	HPRC Release 1	676.63	1,775,915.04
Movi 2 (sampled id 200)	HPRC Release 1	391.62	1,027,844.30
SPUMONI (PML)	HPRC Release 1	121.01	317,596.43
ropebwt3 (SMEM)	HPRC Release 1	68.85	180,707.98
ropebwt3 (SMEM, -l 31)	HPRC Release 1	152.33	399,804.90
ropebwt3 (SMEM, -l 51)	HPRC Release 1	355.74	933,680.66
Movi 1 (PML)*	HPRC Release 2	3,199.82	8,398,421.10
Movi 2 (PML)	HPRC Release 2	3,973.79	10,429,703.80
Movi 2 (blocked deltas)	HPRC Release 2	3,246.35	8,520,790.73
Movi 2 (sampled id 50)	HPRC Release 2	989.31	2,596,593.27
Movi 2 (sampled id 100)	HPRC Release 2	650.69	1,707,825.26
Movi 2 (sampled id 200)	HPRC Release 2	385.14	1,010,856.60
SPUMONI (PML)	HPRC Release 2	113.82	298,733.97
ropebwt3 (SMEM)	HPRC Release 2	48.95	128,475.26
ropebwt3 (SMEM, -l 31)	HPRC Release 2	112.60	295,534.47
ropebwt3 (SMEM, -l 51)	HPRC Release 2	263.22	690,846.29

Table 4. Speed of different methods on different references in terms of both the number of reads queried per second and the number of symbols processed per second. *Note that Movi 1 result on HPRC Release 2 is estimated as the run did not complete successfully due to high memory usage during index construction.
